# Comparative analysis of the efficacy of single-port *versus* traditional multi-port laparoscopic surgery for ovarian cysts : a retrospective cohort study

**DOI:** 10.7717/peerj.20915

**Published:** 2026-03-20

**Authors:** Qiong Xia, Qinghua Zhang

**Affiliations:** Department of Gynecology, Second Affiliated Hospital of Xinjiang Medical University, Xinjiang Medical University, Urumqi, Xinjiang, China

**Keywords:** Ovarian cyst, Single-port laparoscopy, Multi-port laparoscopy, 24-hour Visual Analogue Scale (VAS)

## Abstract

**Objective:**

This study aimed to evaluate the efficacy, safety, and patient-centered outcomes of single-port laparoscopic surgery (LESS) *versus* traditional multi-port laparoscopic surgery for benign ovarian cysts.

**Methods:**

A retrospective cohort study enrolled 260 patients (January 2022–September 2025) divided into single-port group (*n* = 130, transumbilical LESS) and multi-port group (*n* = 130, conventional laparoscopy). Outcomes included perioperative indicators (operative duration, blood loss, time to first flatus/ambulation, hospital stay, 24-hour Visual Analogue Scale (VAS) pain), postoperative day-1 inflammatory markers (C-reactive protein (CRP), IL-6, procalcitonin), preoperative/1-3 month postoperative anti-Mullerian hormone (AMH) (ovarian reserve), hospitalization costs, and abdominal wall appearance satisfaction (POSAS). Propensity score matching (PSM) was used to reduce bias.

**Results:**

After PSM, the single-port group had lower 24-hour VAS scores (2.36 ± 0.86 *vs.* 3.72 ± 1.30, *P* < 0.001), higher abdominal wall satisfaction (97.7% *vs.* 71.5%, OR = 16.8, *P* < 0.001), shorter time to first flatus (15.1 ± 2.9 *vs.* 21.1 ± 4.1 h), ambulation (13.6 ± 2.4 *vs.* 20.9 ± 3.2 h), and hospital stay (3.99 ± 0.60 *vs.* 5.33 ± 1.50 d, all *P* < 0.001). It also had lower costs (17,130 ± 3,793 *vs.* 19,036 ± 4,403 yuan) and inflammatory markers (all *P* < 0.001), and less postoperative shoulder pain (23.8% *vs.* 28.5%, OR = 8.03, *P* < 0.001). Operative duration, blood loss, and 1-3 month AMH showed no inter-group differences (all *P* > 0.05). No conversions, 30-day readmissions, or complication rate differences were observed (*P* > 0.999).

**Conclusion:**

LESS for benign ovarian cysts has comparable safety/efficacy to multi-port laparoscopy, with advantages of less pain, milder inflammation, faster recovery, lower costs, and better cosmetic satisfaction, while preserving ovarian reserve—making it a valuable minimally invasive option.

## Introduction

Ovarian cysts are one of the common gynecological lesions, which can occur at any age, with a higher morbidity in females aged 20 to 50 years (Jiang, Zuo & Zhu, 2023). These cysts have the potential to becoming cancerous and are frequently accompanied by a number of problems. They can be categorized as unilateral or bilateral, solid or cystic, benign or malignant, according on their features. Persistent cysts endanger the health of women and raise the possibility of malignant transformation (Han, Lim & Song, 2022; Nagandla et al., 2021). Surgery is still a viable therapy option at this time (Teede et al., 2023). Laparoscopic surgery is now the recommended treatment for benign ovarian cysts due to the advancement of minimally invasive procedures (Shan, Zhao & Wang, 2024). Single-port laparoscopic surgery (LESS) has become increasingly popular in recent years for treating benign gynecological conditions (Autorino et al., 2013) and has been used extensively for uterine myomectomy, ovarian cystectomy, and treatment of heterotopic pregnancy (Bonollo et al., 2022). LESS offers benefits over typical multi-port laparoscopy, including less trauma, quicker recovery, and less pain. It can also use the umbilicus’s natural folds to create a “scarless” appearance (Massimello & Cela, 2024; Borodulin, Stockwell & Howard, 2020). There is currently a lack of objective assessment data on Chinese patient groups’ satisfaction with the appearance of their abdominal walls. There are currently few comparative research on surgically associated indicators (such the time to resumption of activities and the time to first postoperative flatus). Additionally, neither the short-term effects of single-port and multi-port procedures on ovarian reserve function (anti-Mullerian hormone (AMH) levels) nor the systematic comparisons of their effects on postoperative inflammatory markers (C-reactive protein (CRP), IL-6, and procalcitonin) have been addressed. Inflammatory indicators, changes in AMH levels between 1 and 3 months after surgery, objective aesthetic assessments for abdominal wall appearance, and 24-hour postoperative Visual Analogue Scale (VAS) pain scores were all compared in this study. Clinicians are still primarily concerned with whether LESS is superior to conventional multi-port laparoscopy when treating ovarian cysts. The purpose of this study is to compare the effectiveness of LESS ovarian cystectomy *versus* traditional multi-port surgery in the treatment of ovarian cysts. This study will explore a comparative analysis of the efficacy of single-port laparoscopic ovarian cystectomy for the treatment of ovarian cysts *versus* traditional multi-port surgery.

## Materials and Methods

### Study design

This retrospective cohort study included 260 patients with ovarian cysts who were treated at our hospital between January 2022 and September 2025. Grouping was based on the patients’ informed choice: patients independently selected their surgical technique after being fully educated about the benefits and drawbacks of single-port and multi-port surgery prior to surgery. Patients were split into two groups based on the surgical technique used: 130 patients were in the single-port group, and 130 patients were in the multi-port group. The hospital’s ethics committee approved this study (Ethics Approval Number: KY2023112112). Experienced attending physicians (≥5 years of related surgical experience) detailed to patients and their dependents the differences, advantages and limitations of the two surgeries before they signed informed consent forms. Due to surgical incision and perioperative management differences, blinding patients/surgical teams was unfeasible, but independent nurses (unaware of surgical approach) recorded VAS scores, and patients completed satisfaction questionnaires independently to reduce bias. As indicated in Table 1, statistical analysis revealed no significant differences between the two groups’ baseline data (*P* > 0.05).

**Table 1:** Comparison of general data between the two groups.

	Group		
Characteristic (Mean ± SD)	Multi-port *N* = 130	Single-port *N* = 130	*p*-value
Age	37 ± 11	38 ± 10	0.590
BMI	29.5 ± 3.8	29.1 ± 3.8	0.377
Tumor diameter (cm)	7.43 ± 1.38	7.50 ± 1.26	0.635
Pathology type, n (%)			0.859
1	33 (25.4%)	34 (26.2%)	
2	27 (20.8%)	30 (23.1%)	
3	33 (25.4%)	35 (26.9%)	
4	37 (28.5%)	31 (23.8%)	
Welch Two Sample t-test			
Pearson’s Chi-squared test			

**Notes.**

Endometrial_implantation_cyst:1.

Mature_teratoma:2.

Serous_cystadenoma:3.

Mucinous_cystic_adenosoma:4.

### Criteria for inclusion

1. After preoperative assessment, a benign ovarian cyst was diagnosed, and surgical pathology confirmed this diagnosis.
2. Patients willingly selected either LESS or traditional multi-port laparoscopy for the treatment, and the patients met the surgical indications for laparoscopic ovarian cystectomy.
3. There are no unusual symptoms or indications of infection at the umbilicus.
4. No hormonal medication use throughout the previous six months.
5. There are no serious pelvic adhesions.

#### Criteria for exclusion

1. Possessing obvious reasons why laparoscopic surgery is not appropriate;
2. Possessing cancerous tumors;
3. While pregnant or nursing;
4. When there is a serious organ disease;
5. Suffering from acute or chronic infections or clotting issues.

### Method

(1) Sample size calculations

**Figure 1:**
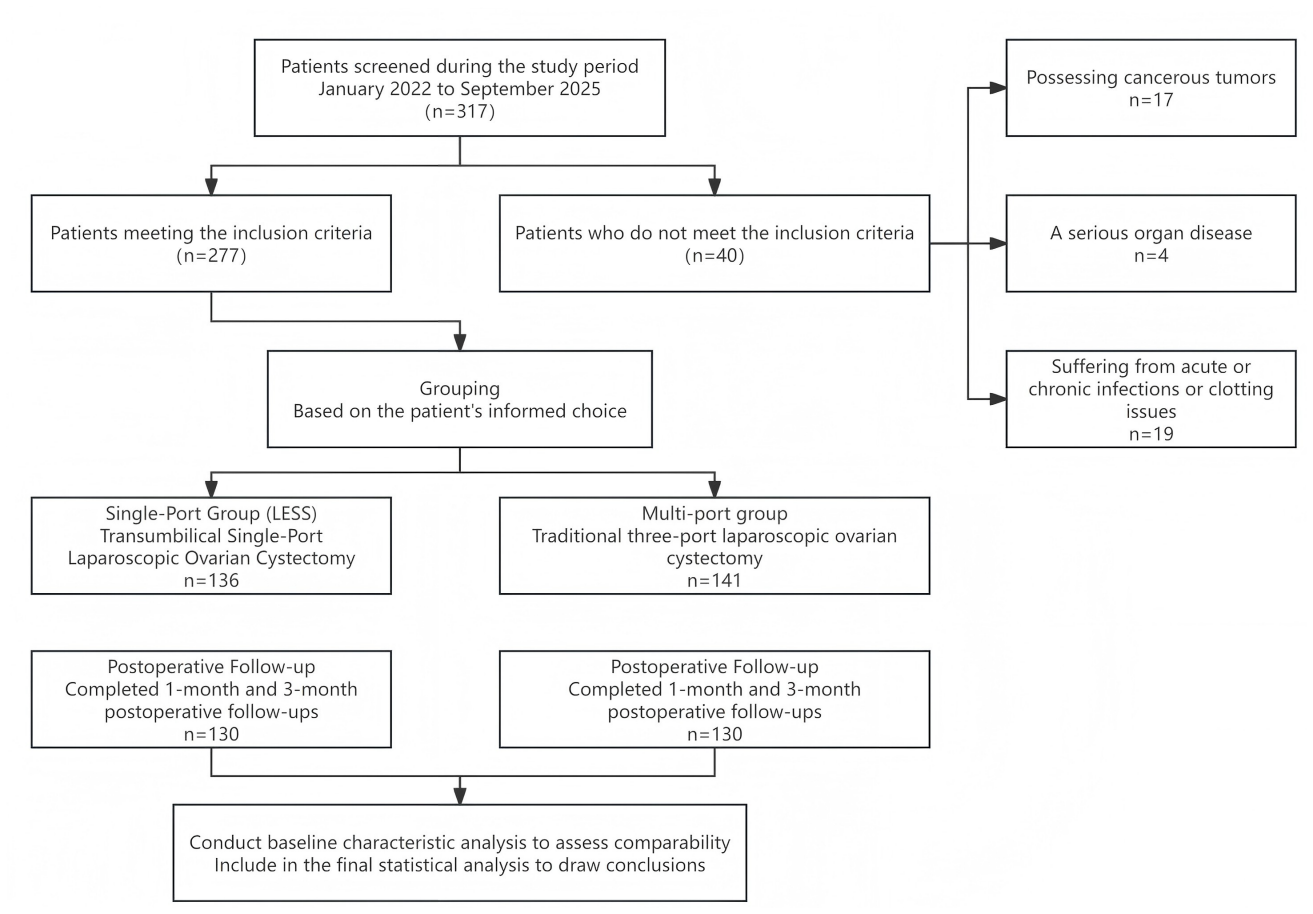
Patient flow diagram.

Software called SPSS 26.0 was used to estimate the study’s sample size. The 24-hour postoperative VAS score served as the main outcome measure. In a pilot study with 30 patients, the single-port laparoscopy group’s estimated VAS ratings were 2.6  ± 1.3, while the traditional multi-port laparoscopy group’s were 3.6 ± 1.3. With a common standard deviation of 1.3 points, the expected mean difference between the two groups was 1.0 points (effect size Cohen’s *d* = 0.77). Using a two-independent-sample *t*-test with *α* = 0.01 (two-sided) and *β* = 0.1 (power = 80%), the necessary sample size was found to be 47 patients each group, for a total of 94 patients. To account for data variability and an anticipated patient dropout rate of approximately 20%, and to ensure statistical robustness, we proactively extended the data collection period and increased the sample size prior to manuscript submission. This expanded the total sample from an initial 118 cases to 260 cases (130 in the single-port group and 130 in the multi-port group) (Fig. 1).

(2) Surgical team

The same group of experienced surgeons who have finished enough cases to become proficient in the learning curve perform all procedures.

(3) Conversion during surgery indications:

If one of the following happens during the procedure, conversion to open surgery or multi-port laparoscopy is recommended: Strong adherences that make safe separation impossible; Severe bleeding that is hard to stop; Malignant tumor suspicion.

(4) Surgical procedure

Both patient groups had general anesthesia with tracheal intubation, were placed in the Trendelenburg lithotomy position, and underwent thorough preoperative examinations. The single-port group patients had transumbilical LESS ovarian cystectomy. Standard skin disinfection was done first, and then the umbilicus was disinfected again with alcohol gauze held in place with tissue forceps. The skin and hypodermis were then cut layer by layer from a 2 to three cm longitudinal incision made in the middle of the umbilicus. Pneumoperitoneum was then established by inserting a disposable incision retractor (HK-TH-60.4TX) into the abdominal cavity and adjusting the pressure to 12–14 mmHg. The cyst’s size, location, and anatomical features were closely monitored under pneumoperitoneum. A bipolar electrocoagulator was used to treat the ovarian surface, and an ultrasonic scalpel or scissors was used to cut the ovarian cortex along its longitudinal axis on the free side of the cyst, all the way to the cyst wall. Using dissecting forceps, blunt dissection was carried out at the junction of the ovarian cortex and the cyst wall. Sharp dissection was used when the ovarian cortex and cyst wall were firmly adhered. Bipolar point-to-point hemostasis was used to control active hemorrhage locations. As an alternative, an aspirator could be used to perform irrigation and aspiration concurrently. Once the bleeding site was well defined, clamping and electrocoagulation could be carried out. Ovarioplasty and suture hemostasis could be carried out following a cystectomy. To attain quick hemostasis, a figure-of-eight stitch was initially positioned at the base of the ovarian cortex before suturing it. After employing a multilayer purse-string approach to stitch the ovary with 3-0 absorbable sutures, the cortex was sutured to return to its normal morphology. A 10 mm trocar was used to insert a specimen bag into the pelvic cavity. After inserting the ovarian cyst into the bag, the mouth of the bag was extracted using a 10-mm trocar. After that, the specimen was taken out and sent for a quick frozen section pathological analysis. Following surgery, the self-made port was taken out, and the wound was cleaned, dressed, and sealed with 3/0 absorbable skin sutures. Depending on the patient’s particular situation, the right supportive treatment measures were given after surgery. The multi-port group’s patients had conventional laparoscopic ovarian cystectomies. An arc incision of about two cm was performed at the inferior edge of the umbilicus after a routine skin cleaning procedure. A laparoscope was positioned, a trocar was introduced, and a pneumoperitoneum was created. Trocars measuring five mm, five mm, and 10 mm were then placed into the patient’s lower abdomen on the left and right sides. The surgical procedures and postoperative care were identical to those of the single-port group.

### Indicators of observation

(1) Surgery-related indicators: Operative time (skin incision to suture completion), intraoperative bleeding (suction volume minus irrigation fluid + gauze weight), time to first ambulation, time to first postoperative flatus, and postoperative hospital stay were recorded by two independent operating room nurses (≥5 years of gynecological laparoscopic experience, not involved in surgery); postoperative shoulder pain incidence (24-hour self-report, “present=1/no=0”, calculated as (affected cases/total cases)×100% (Lee, Ji & Yuk, 2015)) and 24-hour postoperative pain (Visual Analogue Scale (Jiang et al., 2021a), 0=no pain to 10=severe pain) were assessed by a dedicated research nurse (independent of surgical/ward teams) to avoid bias. (2) Inflammatory response: Three milliliters of fasting venous blood were drawn from patients on the first postoperative day. C-reactive protein (CRP), interleukin-6 (IL-6), and procalcitonin levels were measured using the enzyme-linked immunosorbent assay (ELISA) after serum had been separated by centrifugation. (3) Ovarian reserve function: Preoperatively and one and three months after surgery, three milliliters of fasting venous blood were drawn. Following centrifugation, ELISA was used to measure the levels of anti-Müllerian hormone (AMH). (4) Abdominal wall appearance satisfaction: Assessed using the validated Patient and Observer Scar Assessment Scale (POSAS)—total scores categorized as very satisfied (1–3), satisfied (4–6), unsatisfied (7–10); total satisfaction = (very satisfied+satisfied cases)/total cases×100%, with questionnaires completed independently by patients under the dedicated nurse’s guidance (Shan, Zhao & Wang, 2024).

### Analysis of statistics

SPSS version 26.0 was used to handle and analyze the data. Using Q-Q plots and homogeneity of variance tests, all data were evaluated for normality and homogeneity of variance. Measurement data with homogeneity of variance and a normal distribution were reported as mean ± standard deviation ($\bar {x}\pm \mathrm{s}$). For comparisons between groups, independent samples t-tests were utilized, and for comparisons within groups, paired samples t-tests were used. For between-group comparisons, independent samples *χ*^2^ tests were employed, and categorical data were displayed as n. *P*-values below 0.05 were regarded as statistically significant.

## Results

### A comparison of the two patient groups’ surgery-related indicators

There were no statistically significant differences between the two groups in terms of operative time and intraoperative blood loss (Table 2). Regarding postoperative recovery and patient experience metrics, the single-port group significantly outperformed the multi-port group. Specifically, the single-port group demonstrated lower VAS scores at 24 h postoperatively (2.36 ± 0.86 points *vs.* 3.72 ± 1.30 points), and a lower incidence of postoperative shoulder pain (23.8% *vs.* 71.5%) (Table 3). The single-port group also demonstrated shorter times to first postoperative flatus (15.1 ± 2.9 h *vs.* 21.1 ± 4.1 h), time to first ambulation (13.6 ± 2.4 h *vs* 20.9 ± 3.2 h), and postoperative hospital stay (3.99 ± 0.60 d *vs* 5.33 ± 1.50 d) were significantly shorter. Total hospitalization costs were also lower (¥17,130 ± 3,793 *vs* ¥19,036 ± 4,403). Furthermore, no statistically significant differences were observed between groups in the incidence of postoperative complications including lower extremity venous thrombosis, surgical site infection (0.8% in both groups), intra-abdominal hemorrhage, and postoperative fever (both 2.3%) (Table 3).

**Table 2:** Comparison of procedure-related indicators between the two patient groups.

	Group		
Characteristic (Mean ± SD)	Multi-port *N* = 130	Single-port *N* = 130	*p*-value
Operation time (min)	101 ± 9	100 ± 9	0.268
Intraoperative bleeding (ml)	29 ± 15	31 ± 13	0.303
VAS score at 24 h after surgery	3.72 ± 1.30	2.36 ± 0.86	<0.001
Time to the first exhaust after surgery (h)	21.1 ± 4.1	15.1 ± 2.9	<0.001
Time to the first ambulation after surgery (h)	20.9 ± 3.2	13.6 ± 2.4	<0.001
Postoperative hospital stay (day)	5.33 ± 1.50	3.99 ± 0.60	<0.001
Total inpatient costs (yuan)	19,036 ± 4,403	17,130 ± 3,793	<0.001
Welch Two Sample t-test			

**Table 3:** Incidence of postoperative shoulder pain and complication rate in both groups.

	Group		
Characteristic	Multi-port *N* = 130	Single-port *N* = 130	*p*-value
The incidence of postoperative shoulder pain (1 To have, 0 For no), n (%)			<0.001
0	93 (71.5%)	31 (23.8%)	
1	37 (28.5%)	99 (76.2%)	
Lower extremity venous thrombosis, n (%)			>0.999
0	127 (97.7%)	128 (98.5%)	
1	3 (2.3%)	2 (1.5%)	
surgical_infection, n (%)			>0.999
0	129 (99.2%)	129 (99.2%)	
1	1 (0.8%)	1 (0.8%)	
intra_abdominal_bleeding, n (%)			>0.999
0	130 (100.0%)	129 (99.2%)	
1	0 (0.0%)	1 (0.8%)	
postoperative_fever, n (%)			>0.999
0	127 (97.7%)	127 (97.7%)	
1	3 (2.3%)	3 (2.3%)	
Pearson’s Chi-squared test.			

### Inflammatory response comparison of the two groups

There were statistically significant differences between the two groups’ levels of procalcitonin, IL-6, and CRP on the first day after surgery (*P* < 0.05). As seen in Table 4.

**Table 4:** Comparison of inflammatory response on postoperative day 1 between the two groups.

	Group		
Characteristic (Mean ± SD)	Multi-port *N* = 130	Single-port *N* = 130	*p*-value
CRP	27.9 ± 5.9	22.0 ± 4.4	<0.001
IL6	31 ± 6	26 ± 5	<0.001
Procalcitonin	0.24 ± 0.14	0.07 ± 0.04	<0.001
Welch Two Sample t-test			

### Ovarian reserve function comparison of the two patient groups

AMH levels in the two groups did not differ statistically significantly prior to surgery (*P* > 0.05). AMH levels after one and three months after surgery also did not change statistically significantly between the groups (*P* > 0.05). As seen in Table 5.

**Table 5:** Compares the ovarian reserve function of the two patient groups.

	Group		
Characteristic (Mean ± SD)	Multi-port *N* = 130	Single-port *N* = 130	*p*-value
PreoperativeAMH	3.77 ± 0.73	3.66 ± 0.72	0.214
Postoperative 1 monthAMH	3.72 ± 0.71	3.72 ± 0.66	0.983
Three months after surgeryAMH	3.10 ± 1.38	3.18 ± 1.44	0.647
Welch Two Sample t-test			

### Comparison of the two groups’ contentment with the appearance of the abdominal wall

After surgery, the single-port group was more satisfied with the way their abdominal wall looked than the multi-port group (*P* < 0.05) (see Table 6).

**Table 6:** Comparison of satisfaction with abdominal wall appearance of the two groups.

	Group		
Characteristic	Multi-port *N* = 130	Single-port *N* = 130	*p*-value
Subjective satisfaction with scars (not satisfied, 2 satisfied, 3 very satisfied, n (%)			<0.001
1	37 (28.5%)	3 (2.3%)	
2	70 (53.8%)	46 (35.4%)	
3	23 (17.7%)	81 (62.3%)	
Total satisfaction = Very satisfied rate + Satisfaction rate, n (%)			<0.001
0	37 (28.5%)	3 (2.3%)	
1	93 (71.5%)	127 (97.7%)	
Pearson’s Chi-squared test			

**Notes.**

Very satisfied:3.

Satisfied:2.

Unsatisfy:1.

### Propensity score matching (PSM) analysis

To further mitigate potential selection bias and enhance the comparability between the two surgical groups, we performed a 1:1 propensity score matching (PSM) analysis. The matching was based on key baseline covariates, including age, body mass index (BMI), tumor diameter, preoperative pathology type, operative time, and intraoperative blood loss.

**Figure 2:**
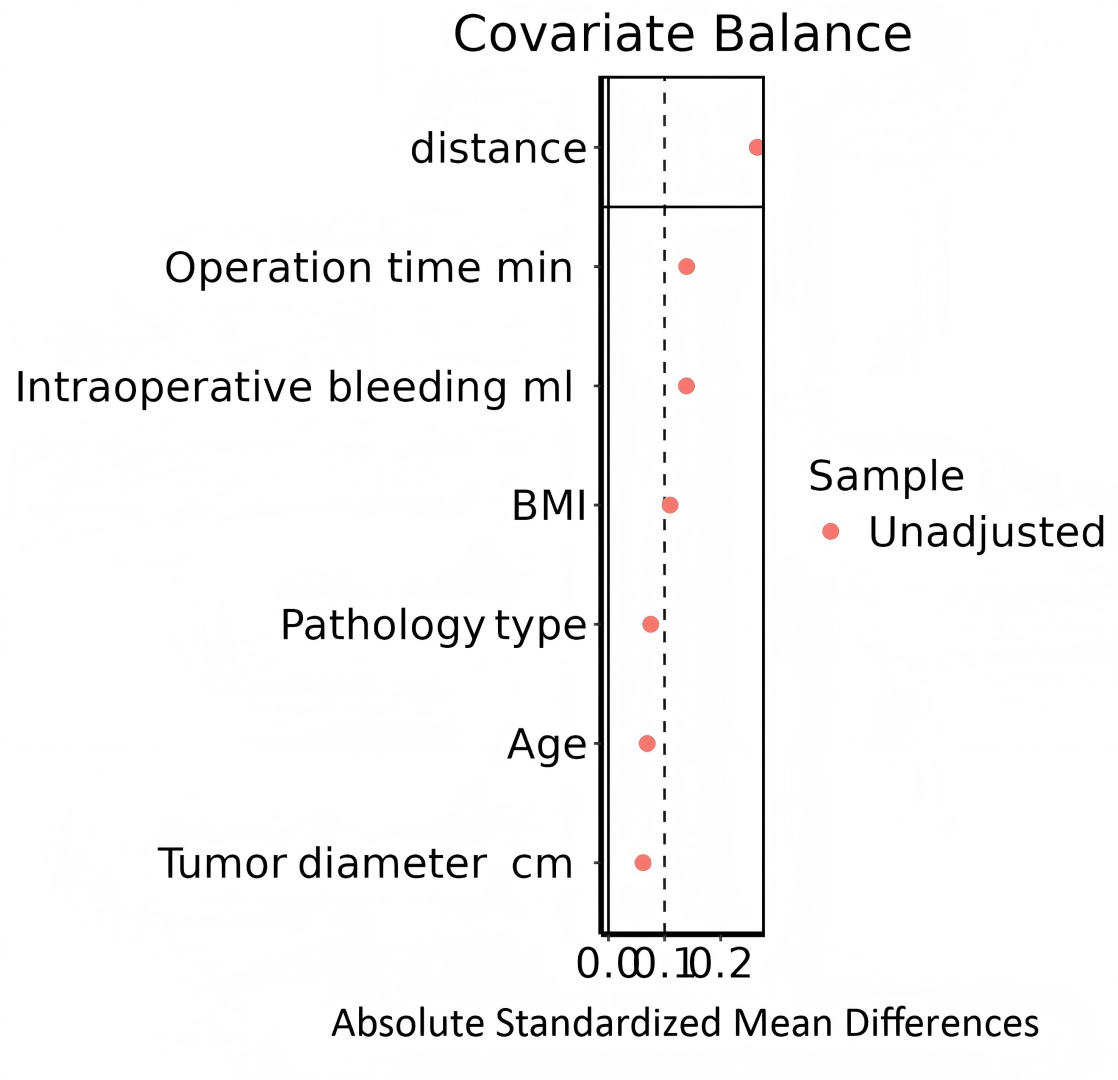
Tendency matching graph.

As detailed in Table 7 and Fig. 2, the baseline characteristics of the two groups were well-balanced after matching, with all standardized mean differences (SMD) reduced to below 0.1, indicating a high degree of comparability between the 130 patients in the single-port group and the 130 patients in the multi-port group.

**Table 7:** Baseline covariates before and after matching.

Variables	Level	Before matching				After matching		
		Multi-port	Single-port	SMD^a^		Multi-port	Single-port	SMD^a^
n		130	130			130	130	
Age (mean (SD))		36.93 (10.78)	37.63 (10.12)	0.069		36.93 (10.78)	37.63 (10.12)	0.069
BMI (mean (SD))		29.51 (3.78)	29.10 (3.77)	−0.110		29.51 (3.78)	29.10 (3.77)	−0.110
Tumor.diameter..cm. (mean (SD))		7.43 (1.38)	7.50 (1.26)	0.062		7.43 (1.38)	7.50 (1.26)	0.062
Operation.time.min. (mean (SD))		100.96 (9.00)	99.73 (8.76)	−0.139		100.96 (9.00)	99.73 (8.76)	−0.139
Intraoperative.bleeding.ml. (mean (SD))		29.32 (15.34)	31.15 (13.16)	0.139		29.32 (15.34)	31.15 (13.16)	0.139
Pathology.type (mean (SD))		2.57 (1.15)	2.48 (1.12)	−0.075		2.57 (1.15)	2.48 (1.12)	−0.075

**Notes.**

a Standardized mean difference.

The analysis of the matched cohorts robustly confirmed the primary findings (Tables 8 and 9). Consistent with the pre-matched results, the single-port laparoscopy group demonstrated statistically significant advantages in multiple short-term recovery outcomes compared to the multi-port group (all *p*-values < 0.001). Specifically, patients in the single-port group had significantly lower 24-hour postoperative VAS scores (mean difference (MD): −1.35, 95% CI [−1.62 to −1.09]), shorter times to first flatus (MD: −5.91 h, 95% CI [−6.77 to −5.04]), earlier ambulation (MD: −7.32 h, 95% CI [−8.01 to −6.63]), and reduced postoperative hospital stay (MD: −1.34 days, 95% CI [−1.62 to −1.07]). Furthermore, the levels of inflammatory markers (CRP, IL-6, and procalcitonin) measured on the first postoperative day remained significantly lower in the single-port group (all *p* < 0.001). The total inpatient costs were also substantially lower for the single-port approach (MD: −1906.18 yuan, 95% CI [−2905.20 to −907.17]).

**Table 8:** Analysis of postoperative efficacy indicators and inflammatory markers between single-port and multi-port laparoscopic surgery (matched data).

**Variables**	**Pairwise comparison**	**Mean difference** ** (95% CI)** ^a^	***p*-value**
VAS score at 24 h after surgery	(Single-port) - (Multi-port)	−1.35 (−1.62, −1.09)	<0.001
Time to the first exhaust after surgery(h)	(Single-port) -(Multi-port)	−5.91 (−6.77, −5.04)	<0.001
Time to the first ambulation after surgery(h)	(Single-port) - (Multi-port)	−7.32 (−8.01, −6.63)	<0.001
Postoperative hospital stay(day)	(Single-port) - (Multi-port)	−1.34 (−1.62, −1.07)	<0.001
Total inpatient costs (yuan)	(Single-port) - (Multi-port)	−1906.18 (−2905.20, −907.17)	<0.001
CRP	(Single-port) - (Multi-port)	−5.86 (−7.13, −4.58)	<0.001
IL6	(Single-port) - (Multi-port)	−4.62 (−5.96, −3.29)	<0.001
Procalcitonin	(Single-port) - (Multi-port)	−0.17 (−0.19, −0.14)	<0.001

**Notes.**

a Based on an ANOVA model.

ANOVA Analysis of Variance CI Confidence Interval SD Standard Deviation

**Table 9:** Logistic regression analysis of postoperative shoulder pain incidence and abdominal wall appearance satisfaction between single-port and multi-port laparoscopic surgery.

**Variables**	**Characteristic**	**OR**	**95% CI**	***p*-value**
The incidence of postoperative shoulder pain (1 To have, 0 For no)	Treatment (Unmatched)			
	Multi-port	–	–	
	Single-port	8.03	4.61, 14.0	<0.001
	Treatment (Matched)			
	Multi-port	–	–	
	Single-port	8.03	4.61, 14.0	<0.001
Total satisfaction	Treatment (Unmatched)			
	Multi-port	–	–	
	Single-port	16.8	5.04, 56.3	<0.001
	Treatment (Matched)			
	Multi-port	–	–	
	Single-port	16.8	5.04, 56.3	<0.001

Logistic regression analysis on the matched pairs further revealed that single-port laparoscopy was associated with a markedly lower incidence of postoperative shoulder pain (odds ratio (OR): 8.03, 95% CI [4.61–14.0]) and a significantly higher overall satisfaction rate with the cosmetic appearance of the abdominal wall (OR: 16.8, 95% CI [5.04–56.3]).

In summary, the PSM analysis strengthens the validity of our results by demonstrating that the superior recovery profiles, reduced inflammatory response, lower costs, and higher patient satisfaction associated with the single-port technique persist in a well-balanced patient cohort, thereby minimizing the influence of confounding factors.

### Distribution of perioperative outcomes, inflammatory markers, and costs between groups

**Figure 3:**
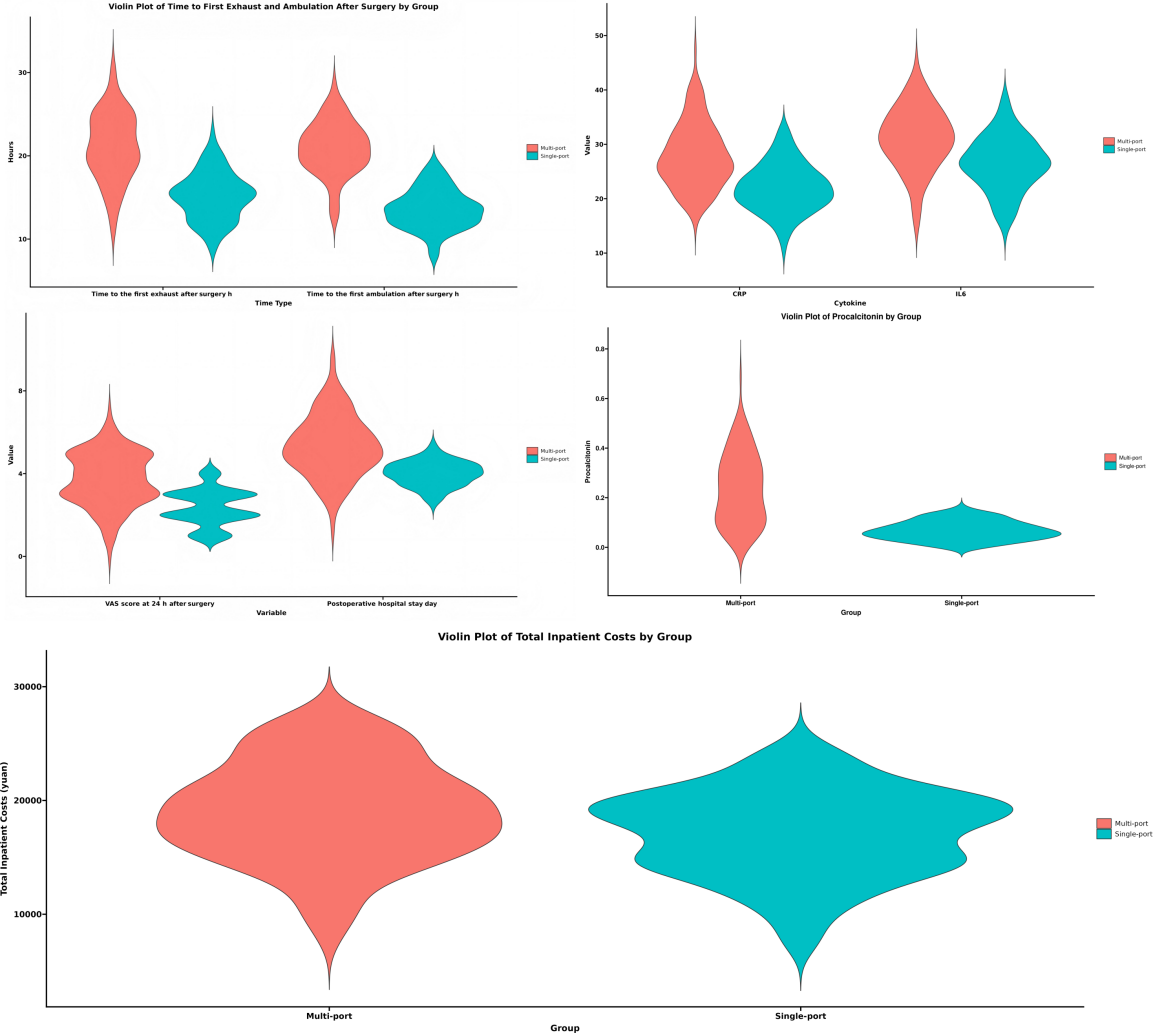
Violin plots.

The violin plots in Fig. 3 visualize the distribution characteristics of key perioperative indicators, inflammatory markers, and costs in multi-port *vs.* single-port laparoscopic surgery groups.

For postoperative functional recovery (top-left panel), the distribution of time to first exhaust and time to ambulation showed a more concentrated trend in the single-port group (teal) compared to the multi-port group (red), suggesting relatively consistent early recovery in the single-port cohort. Regarding inflammatory markers (top-right panel), the distributions of CRP and IL-6 overlapped considerably between the two groups, while IL-8 exhibited a slightly wider spread in the multi-port group.

In terms of patient-reported outcomes and hospital course (middle panels), the VAS score at 24 h (middle-left) showed a more dispersed distribution in the multi-port group, whereas the Postoperative hospital stay presented a narrower concentration in the single-port group. For Proportions (middle-right), the multi-port group had a more concentrated distribution of positive outcomes, while the single-port group showed a flatter, wider spread.

Finally, the total inpatient costs (bottom panel) displayed distinct distribution patterns: the multi-port group had a relatively concentrated, symmetric distribution, while the single-port group exhibited a broader, bimodal-like spread, indicating greater variability in cost expenditure in the single-port cohort.

## Discussion

One of the most prevalent gynecological neoplasms, ovarian cysts can occur in both pre- and post-menopausal patients, and their prevalence is rising in younger populations (Yan et al., 2024). A thorough assessment of the pathogenic kind, patient needs, and technical viability is required before ovarian cysts can be surgically treated. Nowadays, the main surgical techniques are as follows: (1) Laparotomy. Benefits: Makes direct visualization possible; ideal for large cysts (15 cm or larger) or those with a high risk of cancer. Drawbacks include a lengthy hospital stay, a sluggish recovery following surgery, and high levels of invasiveness. (2) Transvaginal cystectomy, or vaginal surgery. Benefits include minimal discomfort, no incision of the abdominal wall, and suitability for posterior cysts without adhesions (Raakow et al., 2020) Drawbacks include a smaller field of vision and a greater likelihood of laparotomy conversion. (3) Standard procedure: traditional multi-port laparoscopy. Benefits: A three-channel procedure that is stable and appropriate for complicated situations (such as severely infiltrative endometriotic cysts). The risk of scarring and prolonged pain following surgery is increased with multiple incisions (Ma et al., 2022). (4) LESS benefit: A single umbilical incision lowers pain scores and produces a favorable cosmetic result. A steep learning curve and the “chopstick effect operative time” are its drawbacks (Zhang et al., 2021). (5) Laparoscopy with robotic assistance Benefit: Suitable for precision suturing, it offers articulated instruments and three-dimensional vision. Low adoption and high cost are drawbacks. Relevant research (Lee, 2021) indicates that LESS is better suited than traditional multi-port laparoscopy for younger patients with low-risk cysts and aesthetic preferences, while multi-port laparoscopy or robotic surgery might be more beneficial in more complicated instances. All of the enrolled cases in this study finished the scheduled surgical procedures.

In comparison to the multi-port group, the single-port group showed reduced 24-hour postoperative VAS pain scores (*P* < 0.05) and higher satisfaction with belly wall cosmesis (*P* < 0.05). Additionally, the single-port group showed a lower incidence of postoperative shoulder pain, a shorter duration of hospitalization overall, a shorter time to first postoperative flatus and first ambulation, and a shorter overall hospital stay (*P* < 0.05). According to these results, LESS has benefits over traditional multi-port laparoscopic surgery for treating ovarian cysts, such as a quicker recovery time, less pain for the patient, and a better abdominal wall appearance after surgery. The umbilicus is selected as the surgical entrance site for LESS ovarian cystectomy. By making use of the umbilicus’s natural folds, this method successfully lowers the development of noticeable surgical scars (Jiang et al., 2021b) and better meets the aesthetic requirements of female patients (Xia et al., 2016). Due to the comparatively sparse vascular, neural, and muscular tissues in the umbilical area (Kaloo et al., 2019), LESS minimizes harm to the arteries, nerves, and muscles of the abdomen wall by reducing the number of ports during surgery. Regarding the cost-effectiveness of single-port laparoscopic surgery (LESS) *versus* traditional multi-port laparoscopic surgery, the significant cost advantage of LESS (*P* < 0.001) stems from comprehensive improvements in medical efficiency driven by its superior short-term outcomes: directly, the shorter postoperative hospital stay (3.99 ± 0.60 days *vs.* 5.33 ± 1.50 days) reduces bed fees, nursing costs, and daily medication expenses, while lower postoperative shoulder pain incidence (23.8% *vs.* 71.5%) and milder inflammatory responses decrease additional costs for analgesics/anti-inflammatory drugs, offsetting the slightly higher cost of LESS-specific disposable retractors; indirectly, earlier recovery allows reproductive-aged patients to resume work earlier, reducing indirect losses from work absence, and high abdominal wall appearance satisfaction (97.7% total satisfaction) avoids potential scar cosmetic treatment costs, while also improving hospital bed turnover for better medical resource utilization. The single-port group experienced significantly less postoperative shoulder pain than the multi-port group (*P* < 0.05). This might be explained by the 2–3 cm umbilical incision made during LESS, which makes it easier to completely expel intra-abdominal CO_2_ gas and avoids negative consequences from leftover gas (Tsiampa et al., 2021). The study’s zero conversion rate highlights the precision of preoperative evaluation and offers proof of the single-port technique’s safety in properly chosen situations. This could, however, restrict how broadly these results can be applied to complicated cysts. To confirm generalizability, inclusion criteria will need to be broadened in further research.

The elevated standard deviation of CRP levels in the multi-port group may be attributed to several factors, such as the multiple incisions (3–4 puncture points) involved in multi-port surgery, the varying degrees of trauma resulting from different incision sizes (5–10 mm), individual differences in inflammatory response sensitivity, and the heterogeneity of baseline CRP levels. Despite a significant absolute difference in CRP values between groups, it is important to note that postoperative CRP elevation following laparoscopy is a normal physiological response. While the inter-group difference in inflammatory markers was statistically significant, its clinical utility requires comprehensive evaluation in conjunction with other signs of infection. This study was limited by the absence of dynamic postoperative CRP monitoring (only a single time point) and the lack of recorded preoperative baseline CRP levels.

Both LESS and multi-port laparoscopic ovarian cystectomy did not significantly impair ovarian reserve function, as evidenced by the fact that neither group’s postoperative AMH levels decreased when compared to preoperative levels (*P* > 0.05). This discovery aligns with the findings published by Tsiampa et al. (2021). Additionally, a study by Schmitt et al. (2024) showed that laparoscopic ovarian surgery was clearly more effective than open surgery at treating ovarian cysts and had a less detrimental effect on postoperative ovarian function. On the other hand, LESS led to a larger decrease of ovarian reserve function in individuals with ovarian cysts, according to research by Lee et al. (2010). Mutual interference may result from the single-port technique, in which all tools enter and exit through a single incision. During surgery, this kind of intervention may unavoidably result in additional harm to healthy ovarian tissue, which would hinder the recovery of ovarian reserve function. Due to the outstanding technical skill of the surgeons performing LESS procedures, accurate cyst dissection in this study reduced injury to normal ovarian tissue. The observed disparity with earlier reports could be explained by this. Furthermore, the difficult problem of specimen retrieval that frequently arises in conventional multi-port laparoscopic procedures was successfully resolved by the bigger umbilical incision. Additionally, the risk of peritoneal spread was decreased by doing away with the requirement for intra-abdominal specimen morcellation, which is more in line with contemporary oncological surgical principles (Tsiampa et al., 2021).

Operative time and intraoperative blood loss did not significantly differ between the single-port and multi-port groups (*P* > 0.05). The single-port group’s operative time was marginally longer, but this difference was not statistically significant. The removal of complicated patients from the study and the surgeons’ completion of their learning curve may be the reasons for this lack of significance. The comparable hemostatic effectiveness of the two surgical techniques may also be the cause of the non-significant variation in blood loss. Additionally, it’s possible that the surgeon’s expertise has a greater impact on blood loss than the actual access technique. Expert surgeons with comparable advanced expertise in the corresponding techniques carried out every procedure in this study. This strategy sought to ensure fair comparability between groups by reducing bias brought on by surgeons’ individual learning curves and skill differences.

There are various restrictions on this study. First, selection bias could be introduced by the non-randomized design. Despite the balance of baseline parameters, unmeasured confounding factors may arise from patients’ self-selection of surgical treatments. Second, the short follow-up period made it difficult to evaluate long-term results and identify modest changes in AMH. These drawbacks imply that rigorous patient selection is necessary and that the alleged benefits of the single-port approach should be regarded cautiously. Additionally, multicenter, prospective, randomized controlled trials are needed to validate its generalizability. Our findings on the safety of single-port laparoscopy (LESS) for benign ovarian cysts—specifically preserved ovarian reserve, superior cosmetic satisfaction, and reduced postoperative pain—are consistent with two recent meta-analyses by Tsiampa et al. (2021) and Bonollo et al. (2022), further validating LESS as a clinically reliable minimally invasive option. Tsiampa et al. (2021) pooled data reported that LESS was associated with non-significant changes in anti-Müllerian hormone (AMH) levels at 1–3 months postoperatively, which directly mirrors our results: AMH levels in both LESS and multi-port groups were statistically comparable at 1 month post-surgery, with no significant divergence at 3 months. This alignment underscores that LESS, when performed by surgeons with proficiency in the technique, avoids excessive manipulation of healthy ovarian stroma,critical for preserving fertility potential in reproductive-aged patients, the primary demographic in both our study (mean age 37–38 years) and the cohort of Tsiampa et al. (2021) (mean age 35–39 years). Regarding cosmetic satisfaction, Bonollo et al. (2022) conducted a systematic review of 13 RCTs (*n* = 1,088) and demonstrated that LESS was associated with a 4.2-fold higher odds of patient-reported ‘excellent’ abdominal wall appearance compared to multi-port laparoscopy. Our data extend this finding: the LESS group in our study had a 16.8-fold higher odds of total satisfaction (very satisfied + satisfied, OR = 16.8, 95% CI [5.04–56.3], *p* < 0.001), with 62.3% of patients reporting ‘very satisfied’ (*vs.* 17.7% in the multi-port group). This amplified effect may stem from our center’s standardized umbilical incision technique, which minimizes visible scarring, a key priority for female patients with benign ovarian cysts, as noted in both our cohort and Ma et al.’s (2022) patient-reported outcome subanalysis. The finding that there is no discernible difference in operating time, however, deviates from the findings of previous meta-analyses (Ma et al., 2022); this disagreement could be due to the technical expertise of our center as well as possible bias in case selection.

## Conclusion

This retrospective cohort study with propensity score matching (PSM) and 260 patients comprehensively compares single-port laparoscopy (LESS) and traditional multi-port laparoscopy for benign ovarian cysts, confirming that LESS is a safe and effective minimally invasive option for carefully selected patients: compared with multi-port laparoscopy, LESS significantly reduces 24-hour postoperative VAS pain scores (mean difference: −1.35, 95% CI [−1.62 to −1.09], *P* < 0.001), shortens time to first exhaust (−5.91 h, *P* < 0.001), first ambulation (−7.32 h, *P* < 0.001) and postoperative hospital stay (−1.34 days, *P* < 0.001), alleviates postoperative inflammatory responses (lower CRP, IL-6, and procalcitonin on postoperative day 1, all *P* < 0.001), reduces total inpatient costs by an average of 1906.18 yuan (95% CI [−2905.20 to −907.17], *P* < 0.001), improves abdominal wall appearance satisfaction (OR = 16.8, 95% CI [5.04–56.3], *P* < 0.001), and lowers postoperative shoulder pain incidence (OR = 8.03, 95% CI [4.61–14.0], *P* < 0.001), while neither approach impairs ovarian reserve (comparable AMH levels preoperatively and at 1/3 months postoperatively, all *P* > 0.05). However, this study has key limitations that must be prominently acknowledged when interpreting results and generalizing findings: the non-randomized design (based on patient informed choice) may introduce selection bias, even with PSM balancing baseline covariates (all post-matching SMD < 0.1), as unmeasured confounders (*e.g.*, psychological expectations, unrecorded subtle preoperative differences) could persist; the 3-month follow-up only assesses short-term outcomes, failing to clarify long-term changes in ovarian reserve, long-term complication rates (*e.g.*, pelvic adhesions), or sustained abdominal wall satisfaction; the single-center setting, strict inclusion/exclusion criteria, and surgery by experienced surgeons (beyond the learning curve) limit external validity, with results potentially inapplicable to complex cases (*e.g.*, huge cysts, severe adhesions) or settings with limited surgical expertise; and the lack of dynamic perioperative inflammatory marker monitoring and preoperative baseline measurements prevents comprehensive evaluation of inflammatory response time courses and full assessment of the clinical significance of intergroup differences. Despite these limitations, LESS still offers an optimized minimally invasive option for eligible benign ovarian cyst patients—especially young women prioritizing cosmetic outcomes and rapid recovery—with future research needing multi-center, large-sample randomized controlled trials (RCTs) to minimize bias, extended follow-up for long-term data, and expanded study populations (including complex cases) to standardize and popularize LESS in gynecological practice.

##  Supplemental Information

10.7717/peerj.20915/supp-1 Supplemental Information 1 Raw clinical data comparing single-hole and multi-hole groups

10.7717/peerj.20915/supp-2 Supplemental Information 2 Raw data on postoperative complications between single-hole and multi-hole groups

## List of Abbreviations

AMH: Anti-Müllerian Hormone
BMI: Body Mass Index
CRP: C-Reactive Protein
ELISA: Enzyme-Linked Immunosorbent Assay
IL-6: Interleukin-6
LESS: LaparoEndoscopic Single-Site Surgery
POSAS: Patient and Observer Scar Assessment Scale
PSM: Propensity Score Matching
VAS: Visual Analogue Scale

## Additional Information and Declarations

### Data Availability

The following information was supplied regarding data availability: The raw measurements are available in the Supplementary Files.
